# Tolerance to Glutaraldehyde in *Escherichia coli* Mediated by Overexpression of the Aldehyde Reductase YqhD by YqhC

**DOI:** 10.3389/fmicb.2021.680553

**Published:** 2021-06-23

**Authors:** Beatriz Merchel Piovesan Pereira, Muhammad Adil Salim, Navneet Rai, Ilias Tagkopoulos

**Affiliations:** ^1^Microbiology Graduate Group, University of California, Davis, Davis, CA, United States; ^2^Genome Center, University of California, Davis, Davis, CA, United States; ^3^Department of Computer Science, University of California, Davis, Davis, CA, United States

**Keywords:** *yqhC*, *yqhD*, glutaraldehyde, resistance, tolerance

## Abstract

Glutaraldehyde is a widely used biocide on the market for about 50 years. Despite its broad application, several reports on the emergence of bacterial resistance, and occasional outbreaks caused by poorly disinfection, there is a gap of knowledge on the bacterial adaptation, tolerance, and resistance mechanisms to glutaraldehyde. Here, we analyze the effects of the independent selection of mutations in the transcriptional regulator *yqhC* for biological replicates of *Escherichia coli* cells subjected to adaptive laboratory evolution (ALE) in the presence of glutaraldehyde. The evolved strains showed improved survival in the biocide (11–26% increase in fitness) as a result of mutations in the activator *yqhC*, which led to the overexpression of the *yqhD* aldehyde reductase gene by 8 to over 30-fold (3.1–5.2 log2FC range). The protective effect was exclusive to *yqhD* as other aldehyde reductase genes of *E. coli*, such as *yahK*, *ybbO*, *yghA*, and *ahr* did not offer protection against the biocide. We describe a novel mechanism of tolerance to glutaraldehyde based on the activation of the aldehyde reductase YqhD by YqhC and bring attention to the potential for the selection of such tolerance mechanism outside the laboratory, given the existence of YqhD homologs in various pathogenic and opportunistic bacterial species.

## Introduction

Glutaraldehyde (1,5 pentanedial) is a biocide that has been commercialized for about 50 years, with broad activity against bacteria, mycobacteria, fungi, viruses, and spores (Russell, 1994). The disinfectant has been widely used in the cosmetic, food, poultry, leather industries, water treatment systems, dentistry, and hospitals (Simões et al., 2011; Vikram et al., 2015). Marketed with name brands such as Glutaral and Cidex, the biocide is commonly used to disinfect medical instruments, especially heat-sensitive, such as flexible and other heat-sensitive endoscopes (Griffiths et al., 1997). Other aldehydes used for disinfection in hospitals include formaldehyde and ortho-phthalaldehyde (Abreu et al., 2013). The mechanism of action of glutaraldehyde in the cells is believed to be through its cross-linking interaction with amino groups of proteins (Russell, 1994).

Bacterial resistance to glutaraldehyde can occur and has been associated with *Mycobacterium* sp. (Griffiths et al., 1997; Manzoor et al., 1999; de Oliveira et al., 2010; Everall et al., 2017) and *Pseudomonas* sp. (Tschudin-Sutter et al., 2011; Vikram et al., 2015). Despite that, the bacterial mechanisms of resistance and tolerance to glutaraldehyde are generally poorly described. The ability of bacteria to withstand various concentrations of this disinfectant was associated with changes in the outer membrane or cell wall (Manzoor et al., 1999), overexpression of efflux pumps (Vikram et al., 2015), and repression of porins (Svetlíková et al., 2009).

**FIGURE 1:**
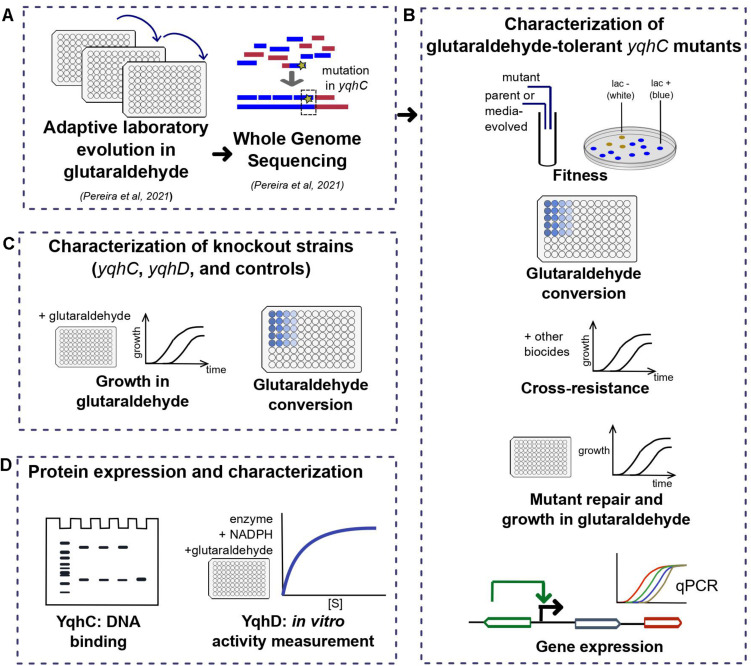
Workflow for the investigation of the *E. coli* tolerance mechanism to glutaraldehyde. In short, **(A)** we have previously detected three tolerant strains after ALE (Pereira et al., 2021) with mutations in different locations of *yqhC*. In this work, we **(B)** characterized the tolerant strains in terms of fitness, growth in glutaraldehyde, cross-resistance to other biocides, expression of *yqhC* and the regulated genes *yqhD* and *dkgA* and glutaraldehyde conversion. To identify the gene responsible for the tolerance phenotype, we **(C)** checked the growth in glutaraldehyde of knockout strains of each gene and various controls and investigated the differences in conversion of glutaraldehyde between mutants, parental strains and knockout strains. Lastly, we isolated the proteins of interest **(D)** and tested for binding to DNA (YqhC) and glutaraldehyde conversion (YqhD).

The gene *yqhC* in *Escherichia coli* regulates the expression of both *yqhD*, a NADPH-dependent aldehyde reductase (Pérez et al., 2008), and *dkgA*, a methylglyoxal reductase (Ko et al., 2005), via activation (Lee et al., 2010; Turner et al., 2011). YqhD was implicated in the detoxification and bacterial survival in aldehydes and other chemicals, such as hydrogen peroxide, paraquat, chromate, potassium tellurite, butanaldehyde, propanaldehyde, acrolein, and malondialdehyde, tert-butylhydroperoxide (Pérez et al., 2008), furfural (Turner et al., 2011), and glyoxal (Lee et al., 2010). We previously identified mutations in *yqhC* after *E. coli* exposure to glutaraldehyde (Pereira et al., 2021). Here, we perform a series of experiments to describe the role of *yqhC*, *yqhD*, and *dkgA* for the survival of *E. coli* in the presence of glutaraldehyde (Figure 1), expanding the knowledge related to the mechanisms of tolerance and resistance to this common disinfectant.

## Results

### The Evolution of *E. coli* in Glutaraldehyde Resulted in Adaptation to the Biocide and Mutations in the Transcriptional Regulator *yqhC*

**FIGURE 2:**
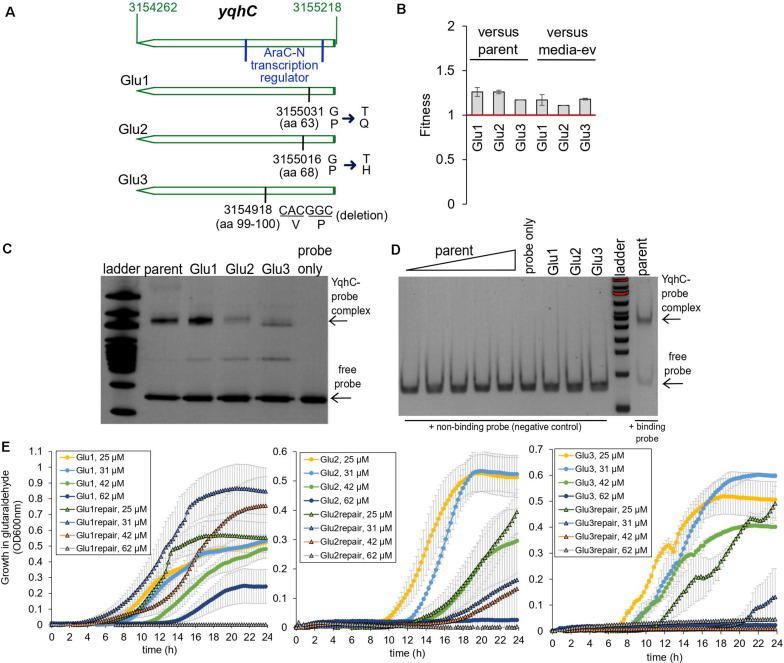
Glutaraldehyde-evolved and tolerant *E. coli* MG1655 strains independently selected for mutations in *yqhC*. **(A)** Position information within the *yqhC* gene of the mutations observed for the evolved strains Glu1, Glu2, Glu3. The bases and amino acids (aa) mutated are indicated. **(B)** Fitness of glutaraldehyde-evolved strains over the parent or media-only evolved strains as measured in competition assays. Fitness above one denotes a fitness advantage. The experiment was performed in duplicate. **(C)** A representative gel-shift (EMSA) assay for each of the YqhC variants. The 100 bp DNA ladder (NEB) was applied to the gel for reference. The bottom arrow points to the free probe, and the top arrow indicates the YqhC protein bound to the DNA probe (promoter region of *yqhD-dkgA* operon). **(D)** A gel-shift (EMSA) assay with a negative-control DNA probe (non-binding DNA sequence) for reference. The concentration of protein for the parent YqhC was varied between 5 and 115 ng/μL. **(E)** Growth curves in M9 media with the presence of glutaraldehyde (concentrations indicated in the graph’s legend) for the evolved strains Glu1, Glu2 and Glu3 and their respective “repair” strains, in which the mutant *yqhC* was replaced with the wild-type version of the gene in the respective background strain.

The exposure of *E. coli* to a sub-inhibitory concentration of glutaraldehyde (30 μM) for 25 days (approximately 500 generations) selected for mutations in the transcriptional regulator *yqhC* in three out of four independently evolved, biological replicates (Pereira et al., 2021). Two mutations (strains Glu1 and Glu2) resulted in single amino acid changes (missense), while strain Glu3 had a six-nucleotide in-frame deletion (Figure 2A). The mutations affected proline residues in all cases. All mutations happened in the N-terminus region of the protein (AraC-N type transcriptional regulator), and the deletion in Glu3 overlapped a predicted binding area for GlaR (Aquino et al., 2017).

In addition to the mutations in *yqhC*, the evolved strains also exhibited mutations in *aes* (Glu1), *uxaA* (Glu2) *icd*, and *rpoA* (Glu3) (Pereira et al., 2021). Despite the additional mutations in all the three glutaraldehyde-evolved strains, the independent selection for *yqhC* mutations in multiple replicates after exposure to glutaraldehyde suggested a primary role of *yqhC* in such an environment. We tested the growth of the evolved strains in the presence of the biocide. The evolved strains exhibited decreased susceptibility to glutaraldehyde compared to non-evolved (parent) and media-evolved (M9-ev) strains. The evolved strains exhibited higher fitness in competition assays against both the parent and media-only evolved strains in the presence of glutaraldehyde (Figure 2B). Such findings indicated an advantage of the selected mutations in *yqhC* for survival in the biocide.

Proline is a unique amino acid residue for protein structure; it supports helical distortions such as kinks and bulges in proteins (Ceruso and Weinstein, 2002). Substitutions of proline residues to other amino acids are often associated with less rigidity of the protein structure (Boone et al., 2015), which may or may not result in loss of function (Hardy and Nelson, 2000). The inclusion of proline residues is a common strategy to improve proteins’ thermal stability (Zhou et al., 2010; Remeeva et al., 2020). Proline substitutions can also change transcription factors’ affinity to different promoters (Fernandez and Plumbridge, 2019). Despite the amino acid changes (Figure 2A) pointing toward a less stable and potentially non-functional protein, the mutated YqhC transcription factors were capable of binding to the *yqhD-dkgA* promoter region in a gel-shift assay (Figure 2C). A negative control DNA probe sequence did not bind to any of the YqhC proteins (Figure 2D). This observation indicated that the protein sequence changes from proline substitutions and deletions did not result in loss of function in our case. On the other hand, we observed that protein overexpression and recovery were lower for the mutated YqhC proteins (data not shown). It is possible that the protein variants, in special Glu2-YqhC and Glu3-YqhC, are less stable than the wild-type YqhC, especially outside the cell environment, which may be a consequence of the proline changes.

We validated the role of *yqhC* mutations vs. other background mutations detected by whole-genome sequencing for glutaraldehyde susceptibility. We replaced the mutated *yqhC* genes in each one of the evolved strains with the wild-type version. The “repair” strains restored a susceptible phenotype and could not grow at the same glutaraldehyde concentrations as the mutants (Figure 2E).

**FIGURE 3:**
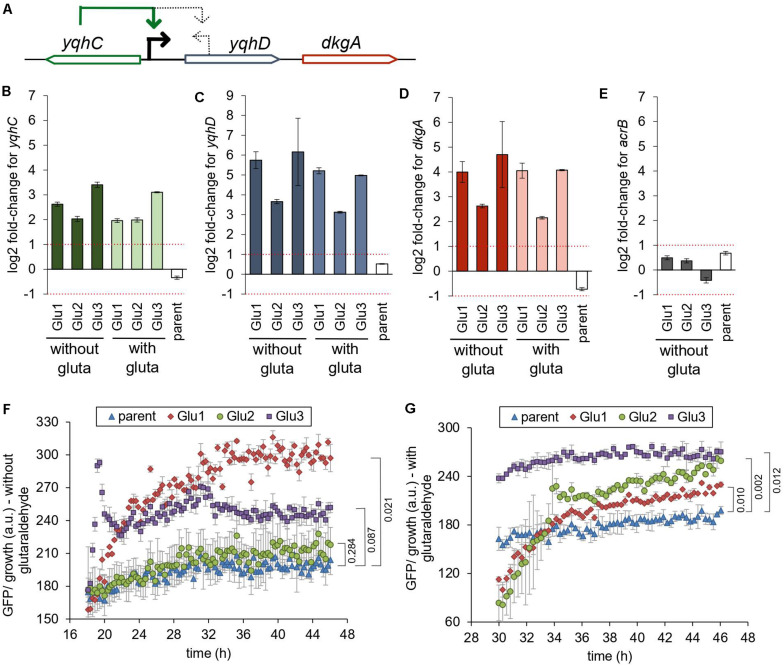
Mutations in *yqhC* are associated with upregulation of *yqhC*, *yqhD*, and *dkgA*. **(A)** The transcriptional regulator *yqhC* activates the aldehyde reductase *yqhD* and the methylglyoxal reductase *dkgA* genes (Lee et al., 2010; Turner et al., 2011). **(B–E)** RT-qPCR results for **(B)** *yqhC* **(C)** *yqhD*, **(D)** *dkgA*, and **(E)** *acrB* expression for the strains evolved in glutaraldehyde, compared to the parent (non-evolved) strain, when grown in the presence or absence of glutaraldehyde. The column “parent” denotes the log2 fold-change between the parent strain in media containing glutaraldehyde compared to media without the biocide. The gene *acrB* was used as a negative control (less than twofold change between evolved and non-evolved strains). The experiments were performed in duplicate, and the error was calculated as described in “Materials and Methods” section **(F,G)**. Expression of GFP under the control of the *yqhC* predicted promoter in each of the indicated strains. **(F)** Minimal media without glutaraldehyde **(G)** Minimal media with glutaraldehyde. The experiments were performed in duplicate, and the standard error was calculated. The *p*-values (one-tailed *t*-test for independent means) for the last time point collected are indicated. gluta refers to glutaraldehyde.

The YqhD protein was implicated in the detoxification and bacterial survival in other chemicals, such as hydrogen peroxide, paraquat, chromate, potassium tellurite, butanaldehyde, propanaldehyde, acrolein, and malondialdehyde, tert-butylhydroperoxide (Pérez et al., 2008), furfural (Turner et al., 2011), and glyoxal (Lee et al., 2010). Despite that, the growth of our mutants in some of such chemicals (potassium chromate, potassium tellurite, and furfural) was not improved compared to the parental strain, in most cases (Table 1). It is possible that other genes or pathways (which are not different between the parental and mutants’ strains) may be relevant for survival in those chemicals, or that the overexpression of YqhD (Figure 3) in our strains was not sufficient to improve growth in the presence of those other chemicals, in the concentrations tested.

**TABLE 1:** Summary of the differences observed for mutant strains compared to the parental strain when grown in minimal media containing the chemicals mentioned.

**Chemical and concentration**	**Glu1**	**Glu2**	**Glu3**
Potassium chromate (0.005–0.01 mM)	Decreased MIC	Slower growth (extended lag)	Decreased MIC
Potassium tellurite (20–60 mg/L)	Decreased MIC	No significant difference	Higher max. growth
Furfural (1–2 mM)	Slower growth (extended lag)	Slower growth (extended lag)	Slower growth (extended lag)

*All strains were tested in triplicates.*

### Mutations in *yqhC* Resulted in Increased Expression of *yqhC*, *yqhD*, and *dkgA*

The gene *yqhC* activates the expression of both *yqhD* and *dkgA* (Figure 3A; Lee et al., 2010; Turner et al., 2011). To determine the effect of the selected mutations of *yqhC* in the known downstream-regulated genes, we measured the expression of *yqhD* (Figure 3C) and *dkgA* (Figure 3D) in the glutaraldehyde-evolved strains with RT-qPCR. All evolved strains exhibited overexpression of both genes (log2 fold-change between 2.15 and 6.16) compared to the parent strain. The overexpression was likely driven by genotype rather than environmental conditions since it was observed regardless of whether strains grew in the presence or absence of glutaraldehyde.

The expression of the unrelated gene for the multidrug efflux protein *acrB*, which was not mutated in any of the evolved strains, was measured on the parent and evolved strains and used as a negative control for the method and to establish a baseline for comparison (Figure 3E). The log2 fold-change for *acrB* was between −1 and 1 for all strains. The parent (non-evolved) strain log2 fold-change in expression of *yqhC*, *yqhD*, and *dkgA* in media with and without glutaraldehyde exhibited values similar to those observed for the control *acrB*.

We also measured the expression of *yqhC* in the evolved strains (Figure 3B). The regulation of *yqhC* and its promoter region exact position is unknown and predicted to overlap with the N-terminal region of the *yqhD* gene (Huerta and Collado-Vides, 2003). The overexpression of the *yqhC* on the mutant strains compared to the parent strain (Figure 3B) suggested that the gene modulates its own expression, as well as the expression of *yqhD* and *dkgA*. We cloned the predicted promoter for *yqhC* before a GFP reporter gene and transformed it into the parent and evolved strains to validate the qPCR results. After growth stabilized and the cells reached the stationary phase, the GFP’s specific expression was higher in the evolved strains compared to the parent, in both the absence (Figure 3F) and presence (Figure 3G) of glutaraldehyde, suggesting a self-regulatory capacity for *yqhC*.

### The Aldehyde Reductase YqhD Has an Exclusive Role for Protection Against Glutaraldehyde

**FIGURE 4:**
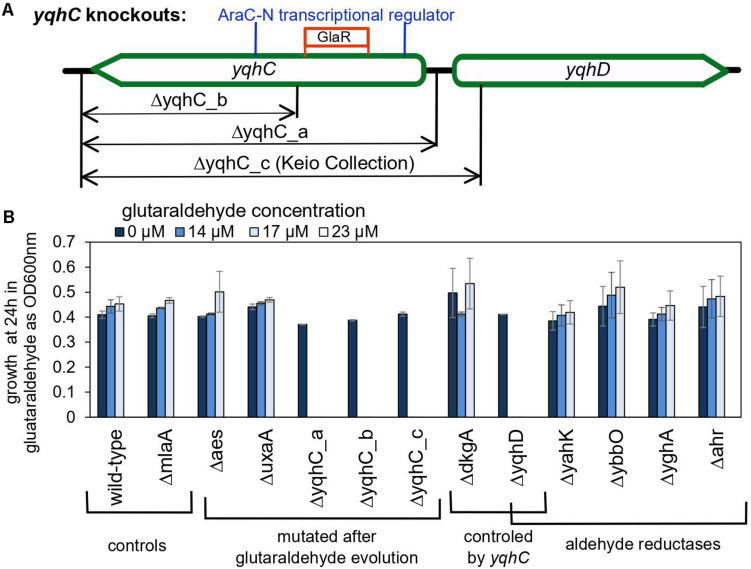
The role of *yqhC*-regulated genes for the survival of *Escherichia coli* in the presence of various concentrations of glutaraldehyde. **(A)** Schematic illustration of the different knockout constructions for *yqhC.* The *yqhC_c* version represents the knockout strain obtained from the Keio Collection (Baba et al., 2006). **(B)** Growth of *E. coli* BW25115 wild-type and knockout mutants (measured as OD600 nm) at 24 h, in the presence of a range of glutaraldehyde concentrations. The genes *aes*, *uxaA* and *yqhC* were mutated in the glutaraldehyde-evolved strains, while *yahK, ybbO, yghA* and *ahr* are genes for other aldehyde reductases in *E. coli* besides *yqhD*. The *mlaA* gene was selected as a negative control to account for a potential effect of the kanamycin cassette over the growth results. Error bars represent standard error of duplicates.

The importance of *yqhD* for bacterial tolerance to the lipid peroxidation-derived aldehydes butanaldehyde, propanaldehyde, acrolein, and malondialdehyde has already been established (Pérez et al., 2008). We sought to verify the role of *yqhC*, *yqhD*, and *dkgA* for the survival of *E. coli* in glutaraldehyde. For that, we evaluated the survival and growth of knockout *E. coli* strains from the Keio Collection (Baba et al., 2006) for each of the genes of interest as well as knockouts for *yqhC* generated using the primers listed in Table 2 (Δ*yqhC_a, ΔyqhC_b*), in minimal media in the presence of glutaraldehyde (Figure 4). The strains Δ*yqhC_a* and Δ*yqhC_b* were constructed by us since the original Keio collection strain knockout for *yqhC* (Δ*yqhC_c*) included partial removal of the N-terminal of the *yqhD* gene sequence (Figure 4A). The knockout strains for *yqhC* and *yqhD* showed higher susceptibility to glutaraldehyde than the wild-type strain. Simultaneously, little to no difference was observed for the knockout strain for *dkgA* and the negative control, the unrelated knockout for *mlaA* (Figure 4B). Our results suggested a role of *yqhD*, but not *dkgA*, for the survival of *E. coli* cells in minimal media in the presence of glutaraldehyde.

**TABLE 2:** Primers and oligos used in this work.

**Primer/oligo ID**	**Strain ID**	**Primer/oligo name**	**Sequence**	**References**
1	Δ*yqhC_a*	yqhC_a_fw	gctcatttctgccaatgtcttgcctatttctccag agtgctggagaaatgattccggggatccgtcgacc	This work
2	Δ*yqhC_b*	yqhC_b_fw	ttatcagaagagattttatgcgcggcggagcggttactcgacgtgatggaattccggggatccgtcgacc	This work
3	Δ*yqhC_a* and Δ*yqhC_b*	yqhC_rv	cgtatttaattcccctgcatcgcccgcattcttgccgcatcttcccccggtgtaggctggagctgcttcg	This work
4	Δ*yqhC_c* (JW5849)	Forward	gaggaatttgttcgcgtaaaccagcgattgcgcctttaccaaacagaatg attccggggatccgtcgacc	Baba et al., 2006
5		Reverse	cgttcccggttgctgtaccgggaacgtatttaattcccctgcatcgcccgtgtaggctggagctgcttcg	Baba et al., 2006
6		Check_yqhc_fw	gccgtaggtaatcaatacgc	This work
7		Check_yqhc_rv	ttgttaggcacgctgtttgt	This work
8		Kan_check_ko	ccgtgatattgctgaagagc	This work
9		Kan_check_ko	gtttctgcggactggctttc	This work
10		Fw-plasmid	atgcgtaaaggagaagaacttttcactg	This work
11		Rv-plasmid	gcgcaacgcaattaatgtaagttagctc	This work
12		Fw-yqhc-prom	cagtgaaaagttcttctcctttacgcatttctccagcactctggagaaatagg	This work
13		Rv-yqhc-prom	gagctaacttacattaattgcgttgcgcggaatttgttcgcgtaaaccagc	This work
14		Seq-primer-promoter	ccgtatgttgcatcaccttc	This work
15		Histag-yqhC	tcagtggtggtggtggtggtgctcgagattcccctgcatcgcccgca	This work
16		Rbs-yqhC	Gaaataattttgtttaactttaagaaggagatatacatatgctacaaaattgcgcacaat	This work
17		Seq-primer-1	cggatatagttcctcctttc	This work
18		Seq-primer-2	tcggtgatgtcggcgatata	This work
19		Primer-probe-1	tacttgctccctttgctggg	This work
20		Primer-probe-2	gcaattttgtagcatttctccagc	This work
21		yqhc repair new rv	atgctacaaaattgcgcacaatcaa	This work
22		yqhc repair new fw	gctgtttgtggtgattaaaaaaaaatactgtaacgcctgaattccggggatccgtcgacc	This work
23		Yqhc-fwd	ctcaataccgccaaattccagc	This work
24		Yqhc-rev	cctgatgatcctgccaattgc	This work
25		Yqhc-seq-fwd	tggcataggtttcgcactcaaac	This work
26		Yqhc-seq-rev	gaaacgtgaagagatttgccgc	This work
27		Negative ctrl probe	cgccgtaggtaatcaatacgcgagcatcgtgaggaatttgttcgcgtaaaccagcgattgcgcctttaccaaacagaatg cgggttggggtgtgcagattaaagttgttcattacttgctccctttgctgggccaatatg	This work
28		yqhD_prt_Fw	gaaataattttgtttaactttaagaaggagatatacatatgaacaactttaatctgcacaccccaacccg	This work
29		yqhD_prt_nostp_rv	tcagtggtggtggtggtggtgctcgaggcgggcggcttcgtatatacggcggctgacatccaacg	This work

*Knockout strains were generated according to the protocol described in*Baba et al. (2006)*and primers 1–9 refer to knockout construction. Primers 1–3 were used to create the indicated ΔyqhC strains. Primers 4 and 5 are mentioned for reference and were originally used by*Baba et al. (2006)*to generate the JW5849 knockout strain used in this work. Primers 6 and 7 were used to amplify the region in which yqhC was replaced by the kanamycin (kan) cassette for sequencing purposes, and the correct insertion of kan cassette was verified with sequencing primers 8 or 9. Primers 10–13 were used to amplify the Pet29b+(GFP) plasmid, and the predicted promoter region of yqhC and primer 14 was used to check for the correct assembly. Primers 15 and 16 were used to amplify the yqhC gene (wild-type and mutants) and primers 17 and 18 were used to verify the correct insertion of the gene into the Pet29b+ backbone. Primers 19 and 20 were used to amplify the probe used for EMSA (DNA binding region for YqhC). Primers 21–26 refer to the construction of gene “repairs.” The kanamycin cassette and wild-type yqhC fragment was amplified from the Keio Collection strain ΔyghB (JW2976)* (Baba et al., 2006) *using primers 21 and 22. Primers 23 and 24 were used to amplify the yqhC gene from genomic DNA from repaired strains to verify the correct insert. Primer 25 was used as a sequencing primer to verify Glu1 and Glu2 yqhC repairs while primer 26 was used as sequencing primer to verify the Glu3 yqhC repair. The negative control (non-binding) DNA probe was amplified from gDNA and is shown in item 27. Primers 28 and 29 were used to amplify yqhD from gDNA for cloning and protein expression.*

We also evaluated knockout mutants for the genes which had SNPs in the evolved strains. The knockout mutants for *aes* and *uxaA* showed no difference in the susceptibility to glutaraldehyde compared to the wild-type control (Figure 4B), reinforcing the exclusive connection between *yqhD* and the susceptibility of *E. coli* cells to glutaraldehyde. The strain Glu3 harbored single nucleotide polymorphisms (SNP) in the genes *icd* and *rpoA* in addition to the deletion in *yqhC*. However, the single knockout mutants for *icd* and *rpoA* cannot grow in minimal media and were therefore not tested in this assay.

Next, we evaluated whether knockouts for other aldehyde reductases of *E. coli* would exhibit a similar effect than observed for *yqhD*. None of the genes classified as aldehyde reductases (*yahK*, *ybbO*, *yghA*, and *ahr*) had an impact in glutaraldehyde survival (Figure 4B), indicating an exclusive role of *yqhD* for protection against the biocide.

### Protection Against the Toxic Chemical Glutaraldehyde Is Mediated by Enhanced Detoxification

**FIGURE 5:**
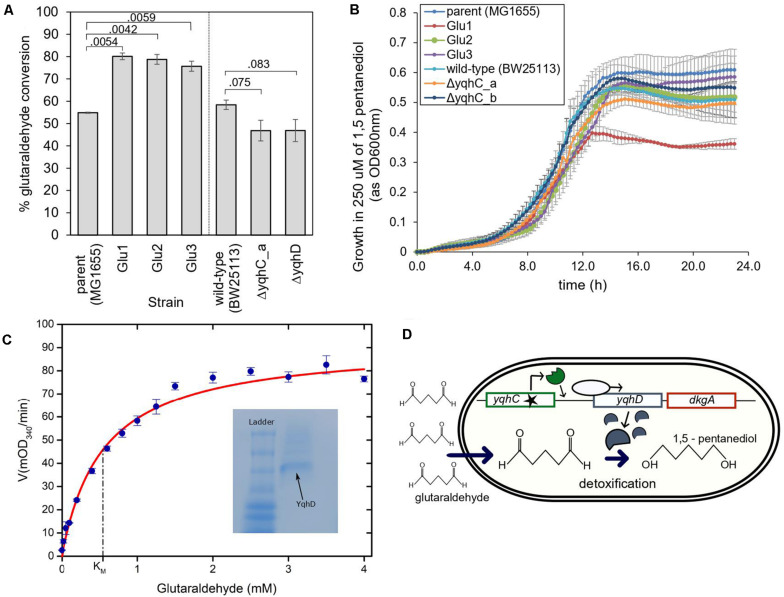
The proposed mechanism for survival of *E. coli* in glutaraldehyde. **(A)** % Glutaraldehyde conversion (degradation) by the evolved strains, knockouts, and their respective parental strains. The standard error is shown for duplicates. The *p*-values are shown above the brackets for each comparison (one-tailed *t*-test for independent means). **(B)** Growth of evolved strains, knockouts, and their respective parental strains in 250 μM of non-toxic 1,5-pentanediol. Such concentration was four to six times higher than the maximum concentration of glutaraldehyde in which evolved strains were able to grow at the otherwise same growth conditions. The standard error is shown for duplicates. **(C)** Michaelis-Menten kinetics of YqhD at different glutaraldehyde concentrations. K_*M*_ of YqhD for glutaraldehyde was calculated to be 0.55 ± 0.09 mM (SEM; *n* = 3 biological replicates). 0.6 mM NADPH and 10 μg of purified YqhD was used. Inset figure shows purified YqhD on native PAGE. **(D)** Mechanism of glutaraldehyde tolerance in *E. coli* MG1655: ALE selected for mutated versions of the transcription factor *yqhC*, which increased expression of the operon containing *yqhD* and *dkgA*. Increased expression of *yqhD* contributed to the survival of *E. coli* cells in the presence of glutaraldehyde due to the conversion of such toxic chemical to the innocuous corresponding alcohol, 1,5 pentanediol, by the aldehyde dehydrogenase YqhD.

We evaluated the conversion of glutaraldehyde in the parent strain, the strains evolved in glutaraldehyde (Glu1, Glu2, Glu3), the knockouts strains for *yqhC*, and *yqhD*, and their correspondent parent strain (*E. coli* BW25113). The evolved strains had improved capacity to detoxify (eliminate) glutaraldehyde from the media, as measured by glutaraldehyde disappearance after 2- and 4-h incubation. All three evolved strains converted over 20% more glutaraldehyde compared to the parent strain (*p* < 0.05) (Figure 5A). A glutaraldehyde-evolved strain without mutations in *yqhC* (Pereira et al., 2021) did not show the same behavior as those studied here, with conversions averaging 36% vs. 75–80% for the strains with *yqhC* mutations, reinforcing the hypothesis for the role of *yqhC* and *yqhD* specifically for glutaraldehyde conversion. In accordance, the knockout strains for *yqhC* and *yqhD* had impaired detoxification capacities, exhibiting conversions about 12% lower than the wild-type strain (*p* < 0.1) (Figure 5A).

YqhD is an aldehyde reductase (Pérez et al., 2008); such enzymes convert aldehyde into their corresponding alcohols. In the case of glutaraldehyde, the corresponding alcohol is 1–5 pentanediol, which, contrary to glutaraldehyde, is non-toxic. We verified the non-toxicity by growing our strains in a 1–5 pentanediol concentration of 250 μM (Figure 5B), which was higher than the glutaraldehyde’s MICs of the strains (between 14 and 125 μM depending on the strain) (Figures 2E, 4B). In addition, we also verified the capacity of the purified NADP-dependent enzyme YqhD to convert glutaraldehyde (K_*M*_ for glutaraldehyde: 0.55 ± 0.09 mM, Figure 5C).

Based on our results, we hypothesize that the protection from glutaraldehyde observed in our evolved strains was the result of YqhD overexpression and mediated by the conversion of glutaraldehyde into the corresponding non-toxic alcohol, 1–5 pentanediol (Figure 5D).

### Genes Homologous to *E. coli’*s *yqhD* Are Present in Bacteria Associated With Outbreaks in the Medical Field

We sought to investigate the prevalence of YqhD and YqhD-like (homologous) proteins in other bacterial species beyond our laboratory model *E. coli* MG1655. A BLASTp (NIH NCBI) search revealed a widespread presence of such homologs in *Escherichia* spp., *Shigella* spp., *Citrobacter* spp., and other closely related Enterobacterium with > 90% protein identity. We further expanded our search to bacteria from *Mycobacterium* spp. Mycobacteria has relevance to human disease. Outbreaks of *Mycobacterium* sp. resistant to in-use concentrations of glutaraldehyde have been reported (Griffiths et al., 1997; Duarte et al., 2009; de Oliveira et al., 2010; Fisher et al., 2012; Burgess et al., 2017), and the mechanism for glutaraldehyde resistance in Mycobacteria is mostly unknown (Everall et al., 2017). When searched using BLASTp (NIH NCBI) against Mycobacteria, the protein sequence for the *yqhD* gene showed similarity to proteins encountered in five isolates of *Mycobacterioides (Mycobacterium) abscessus* (query cover >90% and identity >30%, BLASTp) (Table 3). The NADP(H) dependent BDH family includes the *E. coli* YqhD enzyme and has a preference for substrates with more than three carbons. Although the proteins in the family were annotated as alcohol dehydrogenases, the ability to efficiently detoxify aldehydes (the inverse direction, aldehyde reductase activity) has been demonstrated for enzymes in this group (Pérez et al., 2008; Clarkson et al., 2014; Chung et al., 2015; Kim et al., 2017).

**TABLE 3:** Homology results to *E. coli*’s YqhD in the *Mycobacterium* sp. (BLAST-p, NCBI).

**Sequence ID**	**Query cover**	**Identity**	**Annotation**	**Annotation (region)**	**Most similar to**
SLC18961.1	94%	41.19%	“Iron-containing alcohol dehydrogenase,” “bdhA_2”	BDH (CD08187)	*Bacillus velezensis*
SLC18886.1	89%	39.72%	“Iron-containing alcohol dehydrogenase” “bdhA_1”	YqdH (COG1979)	*Bacillus* sp.
SHS1970.1	99%	39.13%	“1,3 propanediol dehydrogenase” “bdhA_2”	No information	*Bacillus korlensis*
SLL35567.1	94%	39.06%	“Iron-containing alcohol dehydrogenase” “bdhA”	BDH (CD08187)	*Bacillus* sp.
CPW41766.1	97%	36.81%	“Iron-containing alcohol dehydrogenase” “bdhA_3”	BDH (CD08187)	*Enterococcus faecalis*

## Discussion

We previously evolved the gut bacteria *E. coli* in the presence of the biocide glutaraldehyde (Pereira et al., 2021) and observed the independent selection for mutations in the same region of the yet poorly described transcription factor, *yqhC*. Here, we demonstrated that the mutations selected for in the evolved strains increase the *E. coli* survivability in glutaraldehyde by increasing the activation of *yqhD* by the mutated versions of *yqhC*. YqhD is an aldehyde reductase (Pérez et al., 2008), and the tolerance effect is provided by converting the highly toxic glutaraldehyde to the non-toxic correspondent alcohol, 1,5 pentanediol (Figure 5D).

To our knowledge, the three N-terminus mutations that we have observed in the *yqhC* transcription factor gene of *E. coli* and characterized in this work have not been reported before. However, we cannot exclude the possibility that such mutations or other similar mutations in *yqhC* have occurred or were selected for in nature, but not perceived, screened, or reported. In laboratory conditions, exposure of *E. coli* to 10 mM of glyoxal selected for tolerant mutants with changes in the N-terminus of *yqhC* and overexpression of *yqhD* (Lee et al., 2010).

Although we have not measured the product of the glutaraldehyde reduction reaction by the aldehyde reductase YqhD directly, previous work strongly indicates that the YqhD enzyme would convert both aldehyde groups of the glutaraldehyde molecule, resulting in 1,5 pentanediol as a final product, as the enzyme accepts a range of substrates (Jarboe, 2011). As an example, both “malondialdehyde” (Pérez et al., 2008) and “3-HPA” (Li et al., 2008), which have similar structures to glutaraldehyde and 5-hydroxypentanal, respectively, only with 3-carbon instead of 5-carbon chains, are substrates for the YqhD enzyme. Although some intermediate 5-hydroxypentanal, with one aldehyde and one alcohol group, could be present in our case, the enzyme ability to convert a wide range of substrates with similar structures is a strong indication that a complete reduction is happening. In addition to this, 5-hydroxypentanal has a similar toxicity profile to 1,5-pentanediol (non-toxic), contrary to glutaraldehyde. Even a partial reduction by YqhD to 5-hydroxypentanal, if occurring, represents a positive feature for cell survival and tolerance to glutaraldehyde.

We performed the assays utilizing lower than in-use concentrations of glutaraldehyde. The results are nonetheless relevant since resistance in the field may emerge after occasional or accidental exposure to lower than recommended concentrations of biocides, which provide bacteria with suitable environments to adapt and accumulate favorable mutations over time. Glutaraldehyde is an important disinfectant for the health and food sectors (Simões et al., 2011; Vikram et al., 2015). Outbreaks related to bacteria resistant to such biocide have been reported in the field (Griffiths et al., 1997; Duarte et al., 2009; de Oliveira et al., 2010; Fisher et al., 2012; Burgess et al., 2017), but the mechanisms driving resistance are, to date, unclear and possibly driven by the synergetic contributions of multiple resistance-related pathways.

Tolerance to glutaraldehyde was previously associated with changes in expression of porins (Svetlíková et al., 2009) and efflux pumps (Vikram et al., 2015) and with the outer membrane or cell wall composition (Manzoor et al., 1999). In contrast, a large-scale study discarded the presence of a multidrug resistance plasmid as the driver of glutaraldehyde resistance in Mycobacteria (Everall et al., 2017) and couldn’t establish the driver(s) for resistance in the field. In isolates from a glutaraldehyde-resistant *Mycobacterium* spp. from Brazil, the deletion of two alcohol dehydrogenases was one of a few differences observed between the isolated glutaraldehyde-resistant strains and the closest global circulating strain at the time (Everall et al., 2017). The researchers did not further investigate at the time, and the causation effect was not established.

Bacteria can acquire genes through horizontal transfer (Panda et al., 2018). The presence of YqhD and YqhD-homologs in several environmental and clinical isolates implies that a pool of glutaraldehyde-tolerance enzymes is available for selection and transmission. In fact, the YqhD homologs detected in Mycobacterium isolates (Table 3) could have been acquired through horizontal transfer from other species.

Degradation of chemicals by microbial enzymes is a known mechanism of resistance to penicillin (Abraham and Chain, 1940), erythromycin (Barthelemy et al., 1984; Nakamura et al., 2000), and benzalkonium chloride (Zhang et al., 2011; Pereira and Tagkopoulos, 2019) to name a few. There is a plethora of potential enzyme candidates for glutaraldehyde degradation and conversion, which merit further investigation as a bacterial tolerance mechanism to glutaraldehyde. We have shown that other *E. coli* aldehyde reductases besides YqhD do not play a role in glutaraldehyde tolerance (Figure 4B), but a further screening of other specie’s similar enzymes and homologs could improve our current understanding of glutaraldehyde tolerance potential.

Based on the results obtained by us with *E. coli* in a controlled laboratory setting, the observed data on the prevalence of YqhD homologs in various bacterial species, and the genomic information available for resistant *Mycobacterium* spp., it is possible that the presence, absence, or modulation of expression of enzymes such as aldehyde reductases and alcohol dehydrogenases collaborate for the bacterial glutaraldehyde resistance observed in the field. Screening for the presence or overexpression of YqhD homologs in clinical isolates from areas in which glutaraldehyde is used could better shed light on whether such enzymes play a role in bacterial adaptation to the biocide in the field.

## Materials and Methods

### Strains and Solutions

*E. coli* MG1655 was used for the evolution experiments. Strains Glu1, Glu2, and Glu3 described here correspond to glu 1a, glu 3a, and glu 4a, respectively, elsewhere (Pereira et al., 2021). *E. coli* BW25115 wild-type and knockout strains from the Keio Collection (JW5849, JW2978, JW0451, JW5499, JW0465, JW3062, JW2343, JW0317, JW0482, JW2972, JW5761, JW2976) were used (Baba et al., 2006). Chemically competent *E. coli* DH5-alfa was used for the purpose of recovering plasmids, and *E. coli* BLR21(DE3), a protease deficient strain, was used for protein expression. The strains were kept at −80°C with 15% glycerol. Stocks and diluted solutions of glutaraldehyde (Amresco), potassium tellurite monohydrate (Alfa aesar), potassium chromate (Fisher chemical) and 2- Furaldehyde (ACROS organics) were prepared in demineralized water and filtrated with 0.22 um filters before use.

### Adaptive Laboratory Evolution

The evolved strains used in this study were obtained as described elsewhere (Pereira et al., 2021). In short, biological replicates were evolved in 96 well plates for 25 days (500 generations) in Minimal media with 0.4% glucose (M9 glucose) and glutaraldehyde (Amresco). The first inoculum was grown for 12 h in M9 glucose. Every 12 h, approximately 1% of cells were transferred to new wells containing fresh media and the biocide so that cells would remain at exponential (log) phase for most of the duration of the experiment. After growth, glycerol was added to the wells to a final concentration of 15% (v/v), and the plates were stored at −80°C. Next, 2–3 μL of evolved cells were streaked into LB agar plates and grown overnight at 37°C. *E. coli* MG1655 was evolved in M9 glucose media only for 500 generations for comparison. A colony was randomly picked from the plate, grown overnight in M9 glucose media, and stored with 15% glycerol (v/v) at −80°C.

### Growth Curves

Each one of the evolved biological replicates, as well as strain knockouts, “repaired” strains and controls, were tested for susceptibility to glutaraldehyde or other chemicals (potassium chromate, potassium tellurite and 2-furaldehyde) using 96 well-plates (Costar, Corning) containing 193 μL of M9 glucose media, 5 μL diluted glutaraldehyde (or alternative chemical) at various concentrations, and 2 μL of overnight-grown cells with OD_60__0_nm adjusted to 0.1. Plates were incubated at 37°C in a Synergy plate reader for 12–24 h. The assays were performed at least in duplicate, and the error was calculated as the standard deviation divided by the square root of the number of replicates.

### Competition Assays

Competition assays were performed as described elsewhere (Pereira et al., 2021). Cells were grown overnight in 2 mL of M9 glucose, and the OD_60__0_ nm was adjusted to 1.0. For a given assay, 100 μL of an evolved clone and 100 μL of control (either the parent or the media evolved strain) were mixed in a tube containing 10 mL of M9 glucose and glutaraldehyde. The volume was split into three tubes, and a sample was taken from one of the tubes, neutralized with a sodium bisulfite 1% solution, diluted in saline, and plated in X-Gal IPTG LB agar (0.25 mM IPTG Isopropyl β-D-1-thiogalactopyranoside and 40 mg/mL X-gal bromo-chloro-indolyl-galactopyranoside) to determine the cell concentration at time zero. After 24 h, a sample of each of the remaining two tubes was taken, neutralized, diluted in saline, and plated in X-Gal IPTG LB agar. The agar plates were incubated at 37°C, and the cell count (CFU) was determined. Dilutions were determined previously to result in around 50 CFU per plate. To differentiate between the colonies for the evolved clone being tested and the control, one of which had the genotype Δlac (white colonies), the other did not (blue colonies). The Δlac genotype was provided by a loss-of-function SNP on lacY (Pereira et al., 2021). The standard error was calculated as the standard deviation divided by the square root of the number of two replicates. The fitness was calculated according to the recommended formula (Travisano and Lenski, 1996):

$$f\,i\,t\,n\,e\,s\,s=\frac{\ln\left(\frac{A\,1}{A\,0}\right)}{\ln\left(\frac{B\,1}{B\,0}\right)}$$

A(1) = cell concentration for evolved strain after 1 day of exposure to glutaraldehyde.

A(0) = cell concentration for evolved strain at time zero.

B(1) = cell concentration for parent strain after 1 day of exposure to glutaraldehyde.

B(0) = cell concentration for parent strain at time zero.

### DNAseq and Mutation Annotation

The protocol for NGS is described elsewhere (Pereira et al., 2021). In short, the genome DNA was extracted with Wizard Genomic DNA purification kit (Promega) and fragmented using Covaris E220 (microtube AFA fiber snap-cap for 130 μL, peak incident power 140 w, duty factor 10%, 200 cycles per bust, treatment time 70 s). Samples were stored at −20°C, and the KAPA LTP Library preparation Kit for Illumina Platforms (KAPA Biosystems) was used for library construction. The DNA concentration was determined with Qbit or Agilent Bioanalyzer 2100. Final libraries were sequenced with HiSeq 4000 at the DNA Technologies and Expression Analysis Cores (Genome Center, University of California, Davis). The reference genome sequence was NCBI U00096.3. For each sample, the reads were aligned to the *E. coli* K12 (strain MG1655) genome using the short-read alignment tool, Bowtie2 (version:2.3.5.1) (Langmead and Salzberg, 2012). The SNP and short indel mutations were called using VarScan (Koboldt et al., 2009). The criterion for filtering variants was frequency > 49% and *p* < 0.01.

### qPCR

Samples were prepared by mixing the culture with a half volume of cold 5% phenol/ethanol (v/v), following by centrifugation for 10 min at 4,000 rpm and 4°C. The supernatant was discarded, and the cells were stored at −80°C. The RNA was extracted with RNeasy mini kit (Qiagen) and RNAse-free DNAse set (Qiagen). The cDNA was prepared using revert-aid first-strand cDNA synthesis kit (Thermo Fisher Scientific). The qPCR reaction was prepared using Powerup SYBR Green master mix (Applied Biosystems). Plates were sealed with absolute qPCR seal (Thermo Fisher Scientific), spin down, and run using Viaa7 (Applied Biosystems). The gene *ihbF* was used as a housekeeping gene for comparison of the CTs. Each sample (strain plus gene) run in duplicate in the qPCR plate. Results were analyzed using Quantstudio v1.3. The error was calculated with the following formula:

$$error=\sqrt{[\left(\frac{v\,a\,r\,1}{n\,1}\right)+\left(\frac{v\,a\,r\,2}{n\,2}\right)+\left(\frac{v\,a\,r\,3}{n\,3}\right)+\left(\frac{v\,a\,r\,4}{n\,4}\right)}]$$

in which

$$v\,a\,r={\left(s\,t\,d\,e\,v\,o\,f\,r\,e\,p\,l\,i\,c\,a\,t\,e\,s\right)}^{2}$$

and 1,2,3,4 represent the groups formed by the combination of strain and gene (target or housekeeping).

### Chemically Competent Cells and Transformation

Chemically competent cells were prepared from overnight *E. coli* cells, diluted 1:20 in fresh LB media, grown to OD600 nm 0.4–0.9, and chilled in ice for an hour. The cells were pelleted by centrifuging at 3,800 rpm for 10 min at 4°C and dissolved in 0.1 M MgCl_2_. Following centrifugation at 3,800 rpm for 10 min at 4°C, the pellets were resuspended in 0.1 M CaCl_2_ and pelleted again using the same conditions. Finally, competent cells were recovered by resuspending the pellets into 15% glycerol in 0.1 M CaCl_2_ and stored at −80°C until further use. For transformation, 100 μL of competent cells were mixed with 5–10 μL of plasmid, left in ice for about 30 min, and heat-shocked for 1 min 30 s at 42°C. Cells were allowed to grow in 1 mL of fresh LB for 1 h before platting in LB agar containing chloramphenicol. The plates were grown overnight at 37°C, and colonies were picked into fresh liquid LB and grown overnight at 37°C. The plasmids were recovered with GeneJET plasmid miniprep kit (Thermo Fisher Scientific).

### Cloning of yqhC Promoter

The *yqhC* predicted promoter region (Huerta and Collado-Vides, 2003) was amplified from the genomic DNA of *E. coli* MG1655 using primers 12 and 13 (Table 2) and Wizard genomic DNA purification kit (Promega). The pET29b+(GFP) was used as a backbone and amplified using primers 10 and 11 (Table 2). Samples were run in agarose gels and recovered using NucleoSpin gel and PCR cleanup kit (Macherey-Nagel). Assembly was performed with the NEBuilder Hifi DNA assembly master mix (New England BioLabs) according to the instructions. Assembled products were transformed into chemically competent cells prepared previously with heat shock at 42°C for 1 min 30 s. Cells were recovered in LB for 1 h and plated in LB agar containing the appropriate antibiotic. Colonies formed after incubation at 37°C were transferred to liquid LB with antibiotic and incubated at °C overnight. The plasmids were recovered using geneJET plasmid miniprep kit (Thermo Fisher Scientific) and verified for the correct insertion with primer 14 (Table 2).

### Construction of Knockouts

The knockouts for the *yqhC* gene were generated using the pKD46-mediated gene knockout by linear transformation (Baba et al., 2006). In short, the kanamycin resistance gene was amplified from the genomic DNA of the JW2343 knockout strain of the Keio Collection (Baba et al., 2006) with the primers 1–3 listed in Table 2. The amplification product was purified and transformed into cells containing the pKD46 plasmid by electroporation (Micropulser, Biorad). After growth at 37°C for 2–4 h in LB for recovery of cells, the cells were plated in LB with kanamycin and incubated at 40°C for the removal of pKD46. The knockouts were confirmed by PCR and sequencing (primers 6–9, Table 2) and replica-growth in ampicillin.

### Protein Expression and Purification

pET29b+ plasmids were cut with restriction enzymes XhoI and NdeI and assembled with *yqhC* wild-type, *yqhC* mutants or *yqhD* wild-type amplified from genomic DNA using primers 15 and 16 for *yqhC*, and 28 and 29 for *yqhD* (Table 2). *E. coli* BRL21 was transformed with plasmids containing the correct sequences verified with primers 17 and 18 (Table 2). The cells were grown for about 24 h at 250 rpm and 37°C in 5 mL of terrific broth containing kanamycin (25 μg/mL), pelleted at 3,800 rpm for 12 min at 4°C, resuspended in 5 mL of fresh terrific broth containing kanamycin and IPTG (240 μg/mL) and incubated for 4 h at 250 rpm and °C, or 24 h at 16°C. Cells were pelleted at 3,800 rpm for 12 min at 4°C and stored at −20°C or lysed immediately. Lysis was done with gentle movement for 30 min in a mix of 500 μL wash buffer and 500 μL lysis solution (450 μL wash buffer, 250 μL B-PERII- bacterial protein extraction reagent, Thermo Scientific, and 1 mg dry lysis mix). The dry lysis mixture was prepared ahead of time and stored at −20°C and consisted of 10 mg of PMSF, 10 mg of DNAse, and 80 mg of lysozyme. The wash buffer was composed of 20 mM tris-Cl, 5 mM imidazole, 1 mM DTT, 10% glycerol, and 250 mM NaCl. The supernatant containing the proteins was recovered after centrifugation at 14,700 rpm for 30 min at 4°C. The His-tagged YqhC was recovered in microcolumns using 100 μL of Hir-pur Ni-NTA resin slurry (Thermo Fisher Scientific). Following a minimum of six washes, the YqhC was recovered with 300 μL of elution buffer (20 mM tris-Cl, 200 mM imidazole, 1 mM DTT, 10% glycerol, 250 mM NaCl). Protein concentration was determined using the Qubit^TM^ Protein Assay Kit (Thermo Fisher Scientific) and Qubit 2.0 fluorometer.

### Transcription Factor Binding Assay (EMSA)

The purified protein was mixed with probe and binding buffer and incubated for 45 min in ice. The binding buffer consisted of 50 mM NaCl, 20 mM TrisCl, 1 mM DTT, and 10% glycerol. The probe was prepared previously by amplifying genomic DNA using primers 19 and 20 (Table 2) and gel purification. A negative control probe (non-binding DNA) was prepared from the amplification of DNA, and its sequence is shown in Table 2, item 27. After incubation, the mixture was run in native tris-bis gel (4–16%, Thermo Fisher Scientific) with TBE as running buffer for about 2 h at 4°C and 100–150 V. The gel was stained with SyberGreen according to the Electrophoretic Mobility Shift Assay (EMSA) Kit E33075 (Invitrogen) and photographed with Biorad gel doc EZ imager.

### Measurement of the Activity of Purified YqhD

Integrity and purity of purified YqhD protein were checked on a native tris-bis gel (4–16%, Thermo Fisher Scientific) using 1X Native Page Running Buffer (Invitrogen). The gel was stained with Coomassie Plus Protein Assay Reagent (Thermo Fisher Scientific) for 1 h, distained overnight in water, and photographed with Bio-Rad gel doc EZ imager. The enzymatic activity of purified YqhD was determined using the method adapted from Pérez et al. (2008) and measured using a Synergy plate reader at 37°C, in 200 μL of 50 mM phosphate buffer (pH 7.0) supplemented with 0.6 mM NADPH (Sigma-Aldrich), 10 μg of purified YqhD, and 0–4 mM of glutaraldehyde. The K_*M*_ was calculated by OriginPro (OriginLab) using inbuilt Michaelis–Menten function.

### Gene Repair

The *yqhC* alleles (mutated genes) in Glu1, Glu2, and Glu3 strains were reverted to wild-type in each strain by recombination with a linear DNA molecule containing the wild-type *yqhC* allele and an adjacent kanamycin resistance cassette for selection of clones, using the lambda red system as described elsewhere (Pereira et al., 2021). In short, the pkd46 plasmid^1^ was transformed into the strains Glu1, Glu2, and Gu3. The kanamycin cassette and wild-type gene fragment were amplified from the Keio Collection strain Δ*yghB* (JW2976) (Baba et al., 2006) using the primers 21 and 22 (Table 2) and inserted into electrocompetent cells. Colonies that survived in LB kanamycin plates were picked, grown overnight, and stored at −80°C. The correct gene substitution was verified by recovering the genomic DNA with Wizard genomic DNA purification kit (Promega), amplification of the region of interest with primers 23 and 24 (Table 2), and sequencing with primers 25 and 26 (Table 2).

### Electrocompetent Cells and Electroporation

Electrocompetent cells were prepared from fresh overnight cultures grown in ampicillin 50 μg/mL at 30°C. 0.5 mL of overnight were diluted into 50 mL LB, ampicillin 50 μg/mL, and 10 mM L-arabinose and shaken for 3 h at 30°C until the OD_60__0_ nm reached between 0.4 and 0.8. Cells were centrifuged at 4°C, 3,000 rpm for 8 min, and washed sequentially in 50, 20, 5 mL of ice-cold sterile 10% glycerol in deionized water. The last pellet was resuspended in 1 mL ice-cold 10% glycerol. Forty microliter of electrocompetent cells mixed with 2 μL of DNA in a cold 1.5 mL tube were incubated in ice for about 1 min, transferred to a cuvette, and electroporated with Micropulser Electroporator set at Ec1 (Biorad), 1 mL of LB was added, and cells were recovered for 2–4 h at 37°C. Cells were then plated in LB kanamycin.

### Glutaraldehyde Detoxification Assay

The detoxification (disappearance) of glutaraldehyde from the media was measured using the colorimetric aldehyde assay kit blue (MAK140-1KT, Sigma-Aldrich) according to the manufacturer’s instructions. The strains were grown overnight in minimal media, and the OD_600_ nm was adjusted to 0.5 with water. Next, 100 μL of a glutaraldehyde solution were mixed to 1 mL of cells to a final concentration of glutaraldehyde in the tube equal to 220 μM and incubated at 37°C and 250 rpm for 2 and 4 h. Blank tubes were prepared without cells. Next, the tubes were centrifuged at 7,500 rpm for 5 min, and the supernatant was used for the assay. All cells except the three evolved strains with *yqhC* mutations formed a pink-colored pellet after incubation with glutaraldehyde and centrifuging instead of the expected beige. The pink-coloring was not observed when lower concentrations of glutaraldehyde (at least four times lower) or higher concentrations of cells (double the OD_600_ nm) were tested. The visible coloring in the tubes correlated to the presence of residual glutaraldehyde after conversion in the 96-well plates discussed in the results section. Plates were measured at 630 nm after 30 min of incubation in the dark. Conversions were calculated based on aldehyde-equivalent using the aldehyde standard curve (*R*^2^ = 0.9995) generated with the standard solutions provided with the kit.

## Data Availability Statement

The datasets presented in this study can be found in online repositories. The names of the repository/repositories and accession number(s) can be found below: https://www.ncbi.nlm.nih.gov/bioproject/, PRJNA694447.

## Author Contributions

BM and IT conceived the project. BM, NR, and MA performed the experiments, analyzed the data, and created the figures. All authors wrote and revised the manuscript text and figures.

## Conflict of Interest

The authors declare that the research was conducted in the absence of any commercial or financial relationships that could be construed as a potential conflict of interest.

## Footnotes

1 https://www.ncbi.nlm.nih.gov/nuccore/AY048746
